# High-throughput continuous dielectrophoretic separation of neural stem cells

**DOI:** 10.1063/1.5128797

**Published:** 2019-11-13

**Authors:** Alan Y. L. Jiang, Andrew R. Yale, Mohammad Aghaamoo, Do-Hyun Lee, Abraham P. Lee, Tayloria N. G. Adams, Lisa A. Flanagan

**Affiliations:** 1 Department of Biomedical Engineering, University of California, Irvine, Irvine, California 92697-2627, USA; 2 Department of Neurology, University of California, Irvine, Irvine, California 92697-6750, USA; 3 Sue & Bill Gross Stem Cell Research Center, University of California, Irvine, Irvine, California 92697-1705, USA; 4 Department of Anatomy & Neurobiology, University of California, Irvine, Irvine, California 92697-4291, USA; 5 Department of Chemical and Biomolecular Engineering, University of California, Irvine, Irvine, California 92697-2580, USA

## Abstract

We created an integrated microfluidic cell separation system that incorporates hydrophoresis and dielectrophoresis modules to facilitate high-throughput continuous cell separation. The hydrophoresis module consists of a serpentine channel with ridges and trenches to generate a diverging fluid flow that focuses cells into two streams along the channel edges. The dielectrophoresis module is composed of a chevron-shaped electrode array. Separation in the dielectrophoresis module is driven by inherent cell electrophysiological properties and does not require cell-type-specific labels. The chevron shape of the electrode array couples with fluid flow in the channel to enable continuous sorting of cells to increase throughput. We tested the new system with mouse neural stem cells since their electrophysiological properties reflect their differentiation capacity (e.g., whether they will differentiate into astrocytes or neurons). The goal of our experiments was to enrich astrocyte-biased cells. Sorting parameters were optimized for each batch of neural stem cells to ensure effective and consistent separations. The continuous sorting design of the device significantly improved sorting throughput and reproducibility. Sorting yielded two cell fractions, and we found that astrocyte-biased cells were enriched in one fraction and depleted from the other. This is an advantage of the new continuous sorting device over traditional dielectrophoresis-based sorting platforms that target a subset of cells for enrichment but do not provide a corresponding depleted population. The new microfluidic dielectrophoresis cell separation system improves label-free cell sorting by increasing throughput and delivering enriched and depleted cell subpopulations in a single sort.

## INTRODUCTION

The subtle phenotypic differences between cells can be difficult to detect but have big consequences for cell behavior. Separating cells based on their phenotypic differences enables critical experiments aimed at deciphering their biological functions and determining their relevance in disease. Cell separation systems that do not require cell-type-specific labels have a number of advantages. Labels can be limiting since many cells of interest for biological or biomedical applications do not have sufficient markers that distinguish them from other cell types. Labeling of cells could change their biological function, and since this is rarely screened for or tested, incorrect assumptions may be made about the function of labeled cells. Antibodies or labels used for traditional flow cytometry methods bind to cell surface components and could stimulate intracellular signaling cascades. Labeling of intracellular components requires modification of the cell to introduce foreign material that may interfere with normal cellular function. Unlabeled and unmodified cells are also ideal for therapeutic purposes since they require less manipulation that could affect cell phenotype prior to introduction into a patient. Continued development of label-free cell separation technologies will provide much needed alternatives to label-based separation systems.

Many different microfluidic cell separation devices have been developed (Hyun and Jung 2013). Combining multiple separation modalities in microfluidic devices can have advantages over any single approach. Label-free systems include hydrophoresis, in which fluid flow is used to direct cell location in a microfluidic channel, and dielectrophoresis (DEP), in which nonuniform electric fields induce cell movement due to inherent cellular properties (Pethig, 2010; Hyun and Jung, 2013). Hydrophoresis may not have sufficient resolving power to separate cells that are quite similar to each other, particularly cells that are of similar size. DEP can distinguish cells of similar size as long as the cells have distinct electrophysiological properties. For example, similarly sized cells that significantly differ in membrane capacitance can be separated by alternating current (AC) DEP in the frequency range of approximately 1–1000 kHz (Martinsen et al., 2002; Chen and Pohl, 1974; Labeed et al., 2011; Nourse et al., 2014; Simon et al., 2014; Adams et al. 2018). A limitation to DEP-based sorting is that many DEP devices rely on trapping of cells along electrode arrays and release of the isolated cells after washing away nontrapped cells. This “trap and release” mechanism has low throughput due to spatial limits on the number of trapping sites in a device. Combining methodologies such as hydrophoresis and DEP may provide advantages over those of either technique alone.

We developed a microfluidic separation device combining hydrophoretic and DEP modules to create a continuous cell sorter that overcomes the limited throughput of DEP trapping devices. The hydrophoretic module directs all cells to the outer edges of the microfluidic channel. This positions cells for separation by the DEP module, in which the induced DEP force directs targeted cells to the middle of the channel. Channel outlets separately collect two cell populations, those remaining along the outer edges of the channel and those focused to the middle of the channel. Our goal was to create a continuous, rapid, and label-free cell separation system to overcome limitations of sorters using a single separation modality.

## DEVICE DESIGN PRINCIPLES

### Integration of hydrophoretic and DEP modules

**FIG. 1.**
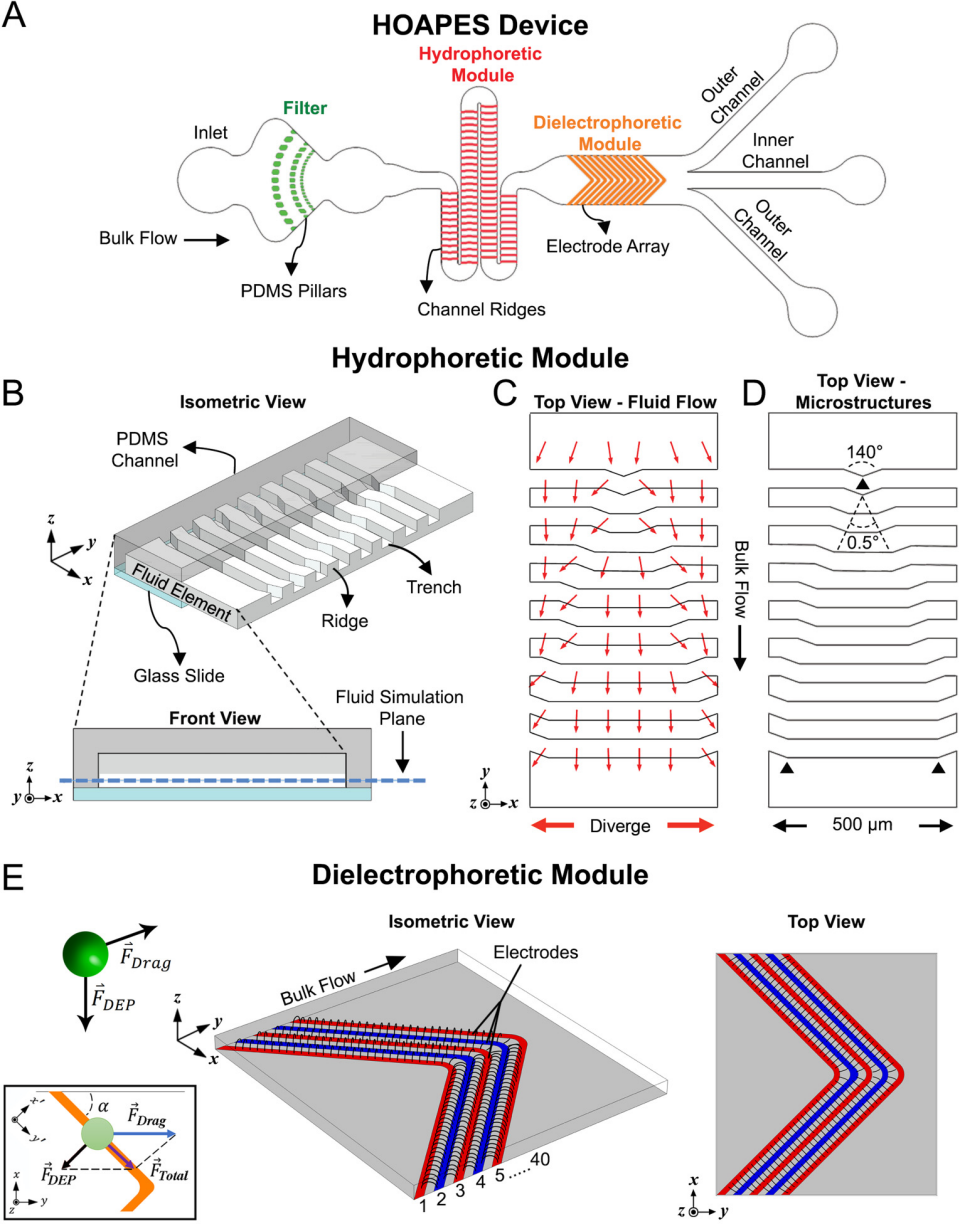
Schematic of HOAPES device with hydrophoretic and DEP modules. (a) A simplified schematic of the HOAPES device shows fluid inlet, PDMS pillars (green) that create a filter to remove cell clumps, the hydrophoretic module (red), DEP module (orange), and a set of 3 outlets (not to scale). (b) An isometric view shows a portion of the hydrophoretic module with PDMS channel (gray), glass slide (blue), and the fluid volume of the channel. Half of the PDMS and glass that make up the channel were removed to depict the details of the fluid within the channel. PDMS microstructures on the ceiling of the channel create fluidic ridges and trenches. A projected front view shows the PDMS channel (dark gray), glass slide (blue), PDMS ceiling microstructures that create fluid trenches (light gray), and fluid (white). The plane used for fluid simulation in panel (c) is denoted by the dashed blue line. (c) A top view of a portion of the hydrophoretic module shows a COMSOL simulation of the diverging fluid flow (red arrows) at the fluid simulation plane. A 10 times scaling factor was applied to the *x* component of the velocity vector to emphasize the direction of the transverse flow. (d) Top view of a portion of the hydrophoretic module shows the ceiling PDMS microstructures, depicting their gradual change in dimension along the channel based on defined diverging angles. (e) A simplified representation of the electric field profile from the 40 electrode array in the DEP module. The isometric view illustrates the electric field distribution in the *z* direction, while the top view illustrates the distribution in the *x*-*y* direction. The green sphere represents a biological cell, and the arrows denote the direction of the drag force and induced DEP force. The inset shows a free body diagram illustrating a cell (green circle) moving along an electrode. The drag force acts in the *y*-direction, the DEP force perpendicular to the electrode, and the combination of the two creates a total force directing cells along the electrodes.

We created a hydrodynamic oblique angle parallel electrode sorter (HOAPES) that incorporates hydrophoresis and DEP in a single platform with a single-step operation. The integrated system performs better than the trap and release methods used in previous DEP devices by continuously sorting cells, minimizing cell-cell interactions and manual operation, and eliminating residual flow. The microfluidic HOAPES device incorporates a filter to remove cell clumps, a hydrophoretic module, a DEP module, and a set of 3 outlet channels [Fig. 1(a)].

Hydrophoresis is the manipulation of suspended particles using microstructure-induced hydrodynamic pressure gradients. Hydrophoresis can be used to direct cells to specific locations in a microfluidic channel without sheath flow. This simplifies device operation since multiple fluidic inlets with balanced flow rates are not needed to create sheath flow to direct cell position in the channel. We utilized a hydrophoretic sheathless aligner working in the laminar flow regime that directs cell location across a wide range of flow rates. This enables efficient and reproducible direction of cells within the channel without costly high-precision instrumentation (Song et al., 2015a). The goal of the hydrophoretic module was to push cells to the channel edges so that all cells would be at a similar position in the channel when encountering the DEP module. The hydrophoretic module working mechanism was evaluated with computational flow simulations, while the microstructure design of the channel was determined with dimensional analysis (also known as the factor label method) (Song and Choi, 2014). Briefly, the hydrophoretic module contains a series of slanted ridged microstructures on the channel ceiling that create a pressure gradient to push cells toward the channel edges. The relationship between cell movement and the dimensionless physical parameters was defined by the following equation (Song and Choi, 2014):

$$L_{p}\propto f\left(\frac{w}{D},\frac{d}{D},\;Re\right),$$ (1)

where $L_{p}$ is the hydrophoretic equilibrium position of the cell, *w* is the width of the fluid channel, *D* is the diameter of the cell, *d* is the depth of the fluid trench, and Re is the Reynolds number (all dimensions listed in equations are depicted in Fig. S1 in the supplementary material). The design constraints for optimal hydrophoretic focusing were reported as (Song and Choi, 2014)

$$\frac{d}{D}\leq 2\;\mathrm{and}\;(h-d)<d,$$ (2)

where *h* is the channel height. Following these design constraints, a simplified computer-aided design (CAD) model with 7 slanted ridged microstructures was designed in SolidWorks and imported into COMSOL Multiphysics to evaluate the flow pattern of the fluid element [Fig. 1(b)]. Fluid flow in the hydrophoretic module was evaluated at a fluid simulation plane 15 *μ*m from the bottom of the channel (blue dashed line in Fig. 1(b), front view). The height of the fluid simulation plane was set at 15 *μ*m from the bottom of the channel since the diverging fluid flow directing cell movement in the channel was maximal at this location (Fig. S2 and Movie 1 in the supplementary material). The maximum transverse flow occurred at every slanted region of the ridged microstructures, generating an overall diverging flow profile that carries the cells toward the channel edges [Fig. 1(c)]. A 10 times scaling factor was applied to the *x* component of the velocity vector in Fig. 1(c) to emphasize the direction of the transverse flow. To take advantage of the maximum transverse flow, we designed a 0.5° angle [Fig. 1(d)] to gradually shift the slanted region toward the channel edges to achieve two focused streams of suspended cells. This design avoided stepwise movement of cells in the hydrophoretic module. Full dimensions of the hydrophoretic module are depicted in Fig. S1 in the supplementary material, and videos show that fluid flow in the module directs cells to the channel edges (Fig. S3 and Movie 2 in the supplementary material).

DEP is one of the most widely used label-free techniques to manipulate cells in microfluidic systems. The induced movement for each cell is highly dependent on the strength and frequency of the applied AC electric field. The specific response of a cell to the applied electric field can be estimated by the complex Clausius-Mossotti (CM) factor. The CM factor, which describes the relationship between the cell and the suspension medium, is defined as

$$CM=\frac{\left(\varepsilon_{cell}^{*}-\varepsilon_{media}^{*}\right)}{\left(\varepsilon_{cell}^{*}+2\varepsilon_{media}^{*}\right)};\;\varepsilon^{*}=\varepsilon +j\sigma /\omega .$$ (3)

Here, the term $\varepsilon^{*}$ is the complex permittivity, ε is the permittivity, $j=\sqrt{-1}$ (imaginary unit), σ is the electrical conductivity, and ω is the angular frequency of the AC electric field. The CM factor can be positive or negative, depending on the relative difference between the polarizability of the cell and the surrounding medium. Since the complex permittivity is a function of the frequency of the applied electric field, different cells with distinct electrical properties may exhibit different responses to the electric field at a particular applied frequency. Furthermore, the DEP force, ${\vec{F}}_{DEP}$, induced on a cell can be estimated by the following equation:

F→DEP=2πR3εmediaRe(CM)∇|E→|2, (4)

where *R* is the radius of the cell, $\varepsilon_{media}$ is the permittivity of the medium, Re(CM) is the real part of the CM factor, and $\nabla |\vec{E}{|}^{2}$ is the gradient of the electric field squared. Hence, when the surrounding medium has greater complex permittivity than the cells, the real part of the CM factor will be less than zero [Re(CM)<0] and the cells will move from high to low electric field regions (negative DEP, nDEP). In contrast, when cells have greater permittivity than the surrounding medium [Re(CM)>0], they will move from low to high field regions of the electric field (positive DEP, pDEP).

We created the DEP module with angled planar interdigitated electrodes in a chevron pattern [Fig. 1(e)]. The goal of the pattern was to pull cells experiencing pDEP to the center of the channel, where they would exit via the inner channel outlet. Cells not in pDEP would remain at the channel edges and exit through the outer channel outlets. The high electric field regions are typically along the electrode edges for planar interdigitated electrodes [Fig. 1(e)]. Therefore, cells experiencing pDEP feel an induced DEP force $\left({\vec{F}}_{DEP}\right)$ perpendicular to the electrodes that pulls the cells toward the electrodes. The pDEP force must be sufficiently strong to attract cells to the electrodes in the presence of the fluid flow. Cells that experience sufficiently strong pDEP to reach the electrodes experience a DEP force perpendicular to the electrode angle [Fig. 1(e), inset]. Hereafter, this force is referred to as ${\vec{F}}_{DEP,xy}$. Coupling the induced DEP force with the viscous drag force $\left({\vec{F}}_{drag}\right)$ parallel to the bulk fluid flow causes the cells to migrate along the electrodes and progressively move down the channel toward the outlets [Fig. 1(e), Fig. S3 and Movie 3 in the supplementary material]. Under normal sorting conditions, the cell's velocity $\left({\vec{v}}_{p}\right)$ is less than or equal to the fluid velocity $\left({\vec{u}}_{f}\right)$; hence, the only drag force acting on the cell is in the *y*-direction from the fluid flow and can be defined by the Stokes drag equation for a spherical cell in laminar flow,

$${\vec{F}}_{drag}=6\pi \eta \vec{v}R;\;\vec{v}={\vec{u}}_{f}-\;{\vec{v}}_{p},$$ (5)

where *η* is the dynamic viscosity of the fluid and $\vec{v}$ is the flow velocity relative to the cell. Thus, the motion of the cells in the *x*-*y* plane is dictated by the vector sum of ${\vec{F}}_{DEP,xy}$ and ${\vec{F}}_{drag}$, and the resultant force $\left(F_{total}\right)$ in the xy coordinate is defined as

$${\left(F_{total}\right)}_{x}={\vec{F}}_{DEP,xy}\cdot \cos(\alpha ),$$ (6)

$${\left(F_{total}\right)}_{y}={\vec{F}}_{drag}-{\vec{F}}_{DEP,xy}\cdot \sin(\alpha ),$$ (7)

where α is the angle of the electrode relative to the wall of the fluid channel. For the cells to move along the electrodes, ${\vec{F}}_{DEP,xy}$ should cancel out the perpendicular component of ${\vec{F}}_{drag}$ with respect to the electrodes. For these calculations, we do not consider additional forces, such as friction from the electrodes or channel surface or gravity or buoyancy. In the $x^{\prime }y^{\prime }$ coordinates [Fig. 1(e), inset],

$$\sum F_{x^{\prime }}=0,$$ (8)

$${\vec{F}}_{DEP,xy}-{\vec{F}}_{drag}\cdot \sin(\alpha )=0\to \;{\vec{F}}_{DEP,xy}={\vec{F}}_{drag}\cdot \sin(\alpha ).$$ (9)

In Eq. (6), if we replace ${\vec{F}}_{DEP,xy}$ with ${\vec{F}}_{drag}\cdot \sin(\alpha )$ derived from Eq. (9), we have

$${\left(F_{total}\right)}_{x}={\vec{F}}_{drag}\cdot \sin(\alpha )\cdot \cos(\alpha ).$$ (10)

The angle of the electrode relative to the channel sidewall was optimized to yield the maximum focusing force, ${\left(F_{total}\right)}_{x}$, toward the center of the channel. Using Eq. (10) to solve for α that yields maximum ${\left(F_{total}\right)}_{x}$ gives

$${\left(F_{total}\right)}_{x}=\frac{\sin\;2\alpha }{2}\cdot {\vec{F}}_{drag},$$ (11)

$$\frac{2{\left(F_{total}\right)}_{x}}{{\vec{F}}_{Drag}}=\sin\;2\alpha ,$$ (12)

$$2\alpha =\frac{\pi }{2};\;\alpha =\frac{\pi }{4}\;\mathrm{or}\;45^{{}^{\circ}}.$$ (13)

The result indicates that the maximum focus occurs when the angle of the electrode is 45° relative to the channel sidewall.

Once we determined that the optimal angle of the electrode relative to the sidewall for cell focusing was 45°, we created a symmetric chevron electrode design resulting in a 90° angle at the electrode tip. The symmetric design doubles the electrode area for focusing cells, thus increasing throughput. The electrode tips were designed to allow the release of cells so they could travel further down the channel. This was accomplished by rounding the tips at the points of the chevron pattern (Fig. S1 in the supplementary material), which effectively decreased the electrode width and increased the gap between the electrodes at the points. The electrodes are 35 *μ*m wide with 35 *μ*m gaps between electrodes (Fig. S1 in the supplementary material). If the electrode points were not rounded, the electrode width at the point would be 50 *μ*m. By rounding the points, the width was decreased to 35 *μ*m, matching the width at the slanted region. The radius of curvature for the inner electrode edge on the rounded tips was 50 *μ*m, while the radius of curvature for the outer edge of the electrode was 85 *μ*m (Fig. S1 in the supplementary material). This differential curvature of the inner and outer electrode edges creates a larger gap of 65 *μ*m between electrodes at the curves. Thus, the slanted electrodes are 35 *μ*m wide with 35 *μ*m gaps, but at the points, the electrodes are 35 *μ*m wide with 65 *μ*m gaps (Fig. S1 in the supplementary material). The electric field strength is lessened at the electrode tips by increasing the gap between electrodes to 65 *μ*m [Fig. 1(e), top view]. The weakened electric field strength at the electrode tips allows cells to release from one electrode to the next and move down the channel. Overall, cells that experience an induced pDEP force will be focused toward the middle of the channel by migrating along the electrodes toward the tips, where they then release to travel further down the channel and exit via the inner channel outlet.

## MATERIALS AND METHODS

### Hydrophoretic oblique angle parallel electrode sorting (HOAPES) fabrication

The HOAPES device is comprised of three main sections: a filter, a sheathless hydrophoretic cell aligner, and a DEP module with oblique parallel electrodes adapted from an earlier design (Lee et al., 2018b). For full dimensions of the HOAPES device, see Fig. S1 in the supplementary material. The channel height is 70 *μ*m, except in the hydrophoretic module where the height varies due to polydimethylsiloxane (PDMS) microstructures on the channel ceiling (see the Device Design Principles section). The device has a single inlet directly followed by an array of 70 *μ*m tall PDMS posts that create a filter to capture cell clumps. In the first row, the posts are 150 *μ*m wide with 100 *μ*m gaps between posts. The second row is 350 *μ*m downstream from the first row, and the posts are 112 *μ*m wide with 75 *μ*m gaps between posts. The third row is 170 *μ*m downstream from the second row, and the posts are 60 *μ*m wide with 40 *μ*m gaps between posts. Details of the hydrophoretic and DEP modules are in the Device Design Principles section. At the end of the DEP module, there are 3 outlet microchannels (one inner and two outer) with 8 *μ*m wide perforations between them to decrease disruption of fluid flow in the event of clogging at the outlets.

**FIG. 2.**
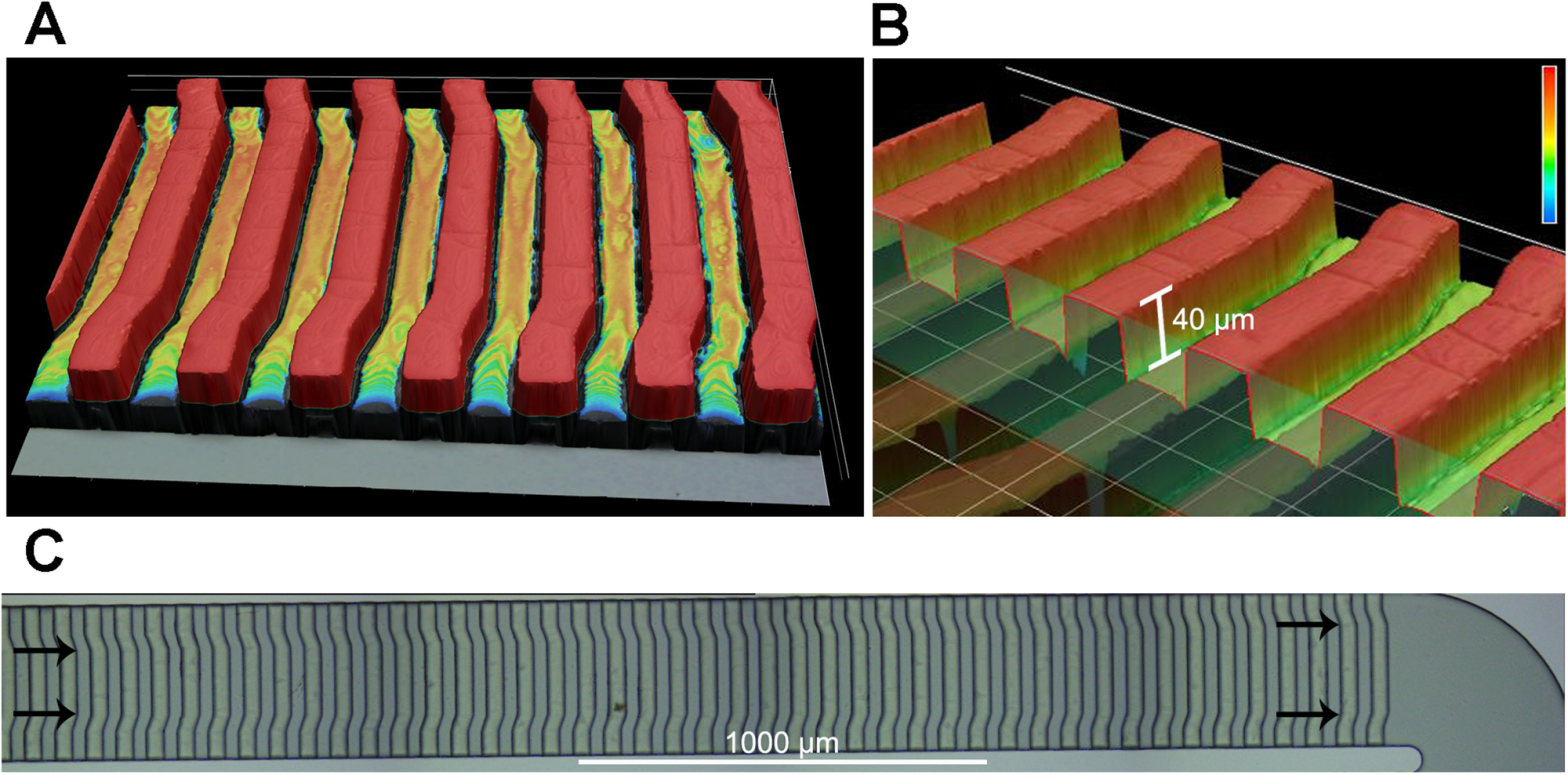
Detail of PDMS microstructures in the hydrophoretic module. Images are from laser scanning confocal microscopy of the silicon mold used to create the PDMS channel for the hydrophoretic module. (a) Raised structures on the silicon mold are highlighted red. (b) A cut away image shows the measured height of the raised structures on the mold. (c) Images of a section of the mold show the gradual change in the angle of the structures (angles indicated by arrows).

The structure of the microchannels was created with two-step photolithography (Choi and Park, 2010). In the first step, a 30 *μ*m thick layer of SU-8 2025 photoresist (MicroChem Corp., Newton, MA, USA) was spin coated onto a silicon substrate, and the first layer photomask was manually aligned and UV cured. In the second step, a 40 *μ*m thick layer of photoresist was spin coated onto the first layer of photoresist, and a second photomask was aligned to the first layer and cured using a mask aligner (Karl Suss MA6 Mask Aligner). Since the PDMS microstructures on the channel ceiling of the hydrophoretic module are critical for cell alignment, we assessed the shape and dimensions of the silicon mold for the hydrophoretic module by laser confocal microscopy (Fig. 2). Laser scanning confocal microscopy was carried out on a 3D laser scanning microscope (Keyence VK-250), which has a nanometer resolution to show surface topology. This analysis confirmed the microstructure shape, gradual change in angle of the structures along the channel, and 40 *μ*m dimension. PDMS was cast onto the mold, cured, and cut into the desired size. Inlet and outlets were punched in the PDMS using a 1.5 mm diameter biopsy punch.

The electrodes were fabricated using standard photolithography techniques previously described (Simon et al., 2014). Briefly, 200 Å titanium followed by 1000 Å gold were coated on standard 25 × 75 mm^2^ glass slides using electron-beam physical vapor deposition. The electrode features were transferred onto the gold-coated slide using a Shipley 1827 positive photoresist (Shipley Company, Marlborough, MA, USA). To assemble the device, the PDMS substrate and the electrode slide were irreversibly bonded after a two-minute oxygen plasma treatment. Finally, 22-gauge solid copper wires were soldered onto the electrode pads for electrical connection.

### Fluid flow and electric field simulations

A finite element computer program (Student Version 5.0, COMSOL Multiphysics, http://comsol.com) was used to simulate both the fluid flow characteristic in the hydrophoretic module and the electric field profile in the DEP module. In both modules, a simplified 3D computer-aided design (CAD) model with fewer repeating elements was created using SolidWorks (2018 student version, http://solidworks.com) and then imported into COMSOL to reduce the processing power required for the simulation (Fig. 1); however, all dimensions of the channel and microstructures were kept consistent with the original design to enable accurate predictions of the fluid flow and electric field distribution.

Hydrophoretic module simulation: The simulation assumed steady state and nonslip boundary conditions at the channel walls. The Navier-Stokes equations for momentum were solved assuming laminar (Reynolds number <2100) and incompressible Newtonian fluid flow (constant density and viscosity). The governing equations in the hydrophoretic module are the continuity and Navier-Stokes equations,

∇⋅u=0, (14)

$$\frac{\partial u}{\partial t}+u\nabla u=-\frac{1}{\rho }\nabla P+\frac{\mu }{\rho }\nabla^{2}u,$$ (15)

where *u* is the velocity vector (m/s), *t* is time, *P* is the pressure (Pa), and *μ*/ρ is the kinematic viscosity (m^2^/s). The DEP buffer calculated density was 1.03 g/cm^3^, and viscosity was 1.1 cP at 25 °C. The flow direction was evaluated at the fluid simulation plane (15 *μ*m from the bottom of the channel) [Fig. 1(b) and Fig. S2 and Movie 1 in the supplementary material].

Electric field simulation: The electric field was simulated using the AC/DC module in COMSOL Multiphysics. The DEP buffer conductivity was set to 100 *μ*S/cm, and the electric potential was set to +3 V or −3 V for each electrode pair.

### Mouse neural stem and progenitor cell (NSPC) culture

CD-1 mice (Charles River) were purchased, selected randomly, and bred as approved by the University of California, Irvine Institutional Animal Care and Use Committee. Dorsal forebrain cortical tissue was dissected from the cerebral cortices of embryonic day 12.5 (E12) mice and placed in a dissection buffer: phosphate buffered saline (PBS), 0.6% glucose, 50 U/ml penicillin/streptomycin. Cortical tissue from multiple embryos within the same litter was pooled, and a subsequent culture from a single litter was considered a biological repeat. The tissue was dissociated using 0.05% Trypsin-Ethylenediaminetetraacetic acid (EDTA) at 37 °C for 10 min. Afterward, trypsin was inhibited using soybean trypsin inhibitor (Life Technologies), and dissociated cells were resuspended in a proliferation medium containing Dulbecco's Modified Eagle Medium (DMEM), 1× B27, 1× N2, 1 mM sodium pyruvate, 2 mM L-glutamine, 1 mM N-acetylcysteine, 20 ng/ml Epidermal Growth Factor (EGF), 10 ng/ml basic Fibroblast Growth Factor (bFGF), and 2 *μ*g/ml heparin. Cells were seeded at 150 000 cells/ml into nontissue culture treated plastic plates and grown as nonadherent spheres. Cell cultures were passaged approximately every 3 days using an enzyme-free NeuroCult Chemical Dissociation Kit (Mouse) (StemCell Technologies). All NSPC cultures were passaged at least once prior to experimental use. Mouse NSPCs were dissociated prior to sorting with nonenzymatic NeuroCult. Dissociated cells were resuspended in a DEP buffer, an iso-osmotic solution consisting of 8.5% (w/v) sucrose, 0.3% (w/v) glucose, and adjusted to a final conductivity of 100 ± 5 *μ*S/cm via addition of RPMI-1640 medium (Flanagan et al., 2008; Lu et al., 2012). DEP buffer conductivity was measured with a conductivity meter (Thermo Orion, Beverly, MA). The final cell concentration was adjusted to 3 × 10^6 ^cells/ml for all experiments.

### NSPC sorting with the HOAPES device

The HOAPES device was placed on a hot plate set at 150 °C for 30 min to sterilize and remove moisture. Fluid flow to the device was driven by a syringe pump (Harvard Apparatus PicoPlus, Holliston, MA) pushing a 1 ml syringe with 1.5 mm outer diameter Tygon® tubing connected to the device inlet. To remove bubbles from the microchannels and sterilize, 70% ethanol was pumped into the device at 20 *μ*l/min. Filtered milliQ (MQ) H_2_O was then flowed into the device at 20 *μ*l/min for 15 min to wash away all ethanol. Bovine serum albumin (BSA, 5%) diluted in filtered MQ H_2_O was then washed through the device for 15 min at 10 *μ*l/min to coat the walls of the microchannels, preventing cell sticking. BSA was washed away with 100 *μ*S/cm DEP buffer at 20 *μ*l/min for 15 min. The device was then mounted on an upright Olympus microscope (model BX41) with bright field objectives and connected to a function generator (AFG320, Tektronic, Beaverton, OR). A commercial dSLR camera (Canon model EOS Rebel T2i) was attached to the microscope to record videos and monitor sorting.

The appropriate sorting frequency was optimized for each batch of cells by generating a presort focusing curve. NSPCs were loaded into the HOAPES device at 3.5 *μ*l/min flow rate, and the DEP module was actuated at 6 V peak-to-peak (Vpp) and the frequency swept from 10 kHz to 1000 kHz. The number of cells focused to the center, inner outlet channel and unfocused to the outer channels was determined for each frequency. The number of cells in the inner channel was divided by the total number of cells to determine the percentage of cells focused at each frequency. The focusing curve (percentage of cells focused at each frequency) was then used to determine the appropriate frequency for sorting in order to collect the desired percentage of focused cells.

NSPC sorting was carried out in batches to minimize cell settling. Cells were loaded into the device by attaching Tygon tubing to the end of a 1 ml syringe. Cells were not directly loaded into the syringe since this resulted in cell settling. The syringe was primed with a DEP buffer, and then 30 *μ*l of cells in the DEP buffer was drawn into the tubing. The tubing was attached to the channel inlet, with care taken to avoid air bubbles by merging the droplet at the end of the tubing with a convex droplet of fluid at the channel inlet. The tubing was mounted vertically to minimize the effects of cell settling. The process was repeated to load additional 30 *μ*l batches of the cell solution into the device as needed. The electrodes were actuated at the sorting frequency, and the syringe pump turned on to induce flow of cells into the channel. In all sorts, cells were exposed to electric fields for less than 1 min. At the end of each run, focused cells from the inner outlet and unfocused cells from the outer outlets were collected and placed in separate Eppendorf tubes. Additional runs were completed at a control frequency (1 MHz) in which the majority of the cells focused to the inner outlet. If some cells remained unfocused, they were collected from the outer outlets and mixed with the focused cells, creating an unsorted control sample. Two other control samples were collected: cells that remained in the proliferation media (media control) and cells that were incubated in the DEP buffer until the end of the sort (DEP buffer control). At the end of each sort, 50 000 cells from each condition (controls, focused, unfocused) were evenly plated onto 12 mm laminin-coated coverslips in the proliferation media. Cells were then differentiated and immunostained to assess astrocyte formation (described below). Actual device throughput was calculated from cell recovery data from sorting experiments, while theoretical device throughput was calculated from the flow rate in the device and cell concentration used for sorting and assumed no cell losses and steady state separation.

### Z-projections of cells in the device

Videos were used to highlight trajectories of mouse NSPCs moving through the device to determine cell locations across multiple frequencies. Videos were stacked in ImageJ using the standard deviation of the intensity (maps the change in intensity from one frame to another) to create images. Each stacked Z-projection image was generated from 30 s of video, and the signal intensity across the channel in each image was measured using ImageJ.

### NSPC differentiation and immunostaining

NSPCs were plated as adherent cultures for differentiation. HCl-washed German glass coverslips (Assistant/Carolina Biological Supply, Burlington, NC) were pretreated with poly-D-lysine (40 *μ*g/ml in milliQ H_2_O) for 5 min then coated with laminin (20 *μ*g/ml in Eagle's Minimum Essential Medium (EMEM)) at 37 °C for 4–24 h prior to cell adhesion. NSPCs were seeded onto the laminin-coated coverslips in the proliferation medium. After 24 h, the proliferation medium was removed and replaced with the differentiation medium (same components as the proliferation medium but excluding EGF, bFGF, and heparin) to induce differentiation. NSPCs were differentiated into astrocytes in these conditions for 3 days. After differentiation, cells were immunostained with anti-glial fibrillary acidic protein (anti-GFAP) monoclonal antibody (Sigma Aldrich, Cat No. G3893, clone GA5) at 1:200 and stained with a secondary antibody (Alexa 555, Jackson ImmunoResearch) at 1:200 as previously described (Flanagan et al., 2008; Labeed et al., 2011). Cells counted as astrocytes exhibited typical astrocyte morphologies and a filamentous pattern of GFAP reactivity in the cytoplasm. Controls included cells stained with secondary antibodies only (negative controls) and appropriate subcellular localization of antibody signal (cytoskeletal for GFAP intermediate filament protein). Cells were imaged with an inverted Nikon-TE fluorescence microscope. Three to 5 randomly selected fields for each 12 mm coverslip were selected for quantitation. From these fields, the number of GFAP+ cells and the number of nuclei were counted using ImageJ. The percentage of GFAP+ cells was calculated for each collected sample.

### Statistical analysis

Statistical analysis was completed using one-way ANOVA with a Tukey *post hoc* test for multiple comparisons for samples with n = 3 or more biological repeats. Biological repeats are listed as “n” in figure legends.

## RESULTS

### Cells for testing the HOAPES device

We tested cell sorting in the new HOAPES device with mouse neural stem and progenitor cells (NSPCs). These cells form distinct differentiated cell types in the central nervous system, particularly astrocytes and neurons. Cultures of mouse NSPCs contain cells that are biased toward forming astrocytes and others that are neuron-biased (Flanagan et al., 2008; Labeed et al., 2011). These astrocyte- and neuron-biased cells do not differ significantly in size and express similar markers, making them difficult to separate from each other using traditional means (Flanagan et al., 2008; Labeed et al., 2011). However, astrocyte- and neuron-biased cells do differ in the electrophysiological property membrane capacitance (Flanagan et al., 2008; Labeed et al., 2011). Differences in membrane capacitance are sufficient to enable the DEP-based separation of astrocyte-biased and neuron-biased cells without the use of cell-type-specific labels (Nourse et al., 2014; Simon et al., 2014; Adams et al., 2018; Yale et al., 2018). DEP is generally not toxic for mouse NSPCs (Lu et al., 2012). However, cells exposed to certain frequencies in DEP for times longer than 5 min show a decrease in viability (Lu et al., 2012). Thus, a continuous DEP sorting system in which cells are exposed to electric fields for seconds rather than minutes (as needed for DEP trapping devices) would be advantageous. The specific properties of mouse NSPCs and the desire to decrease their exposure to electric fields make them an ideal test case for continuous label-free DEP sorting.

### Functional analysis of the HOAPES hydrophoretic and DEP modules

**FIG. 3.**
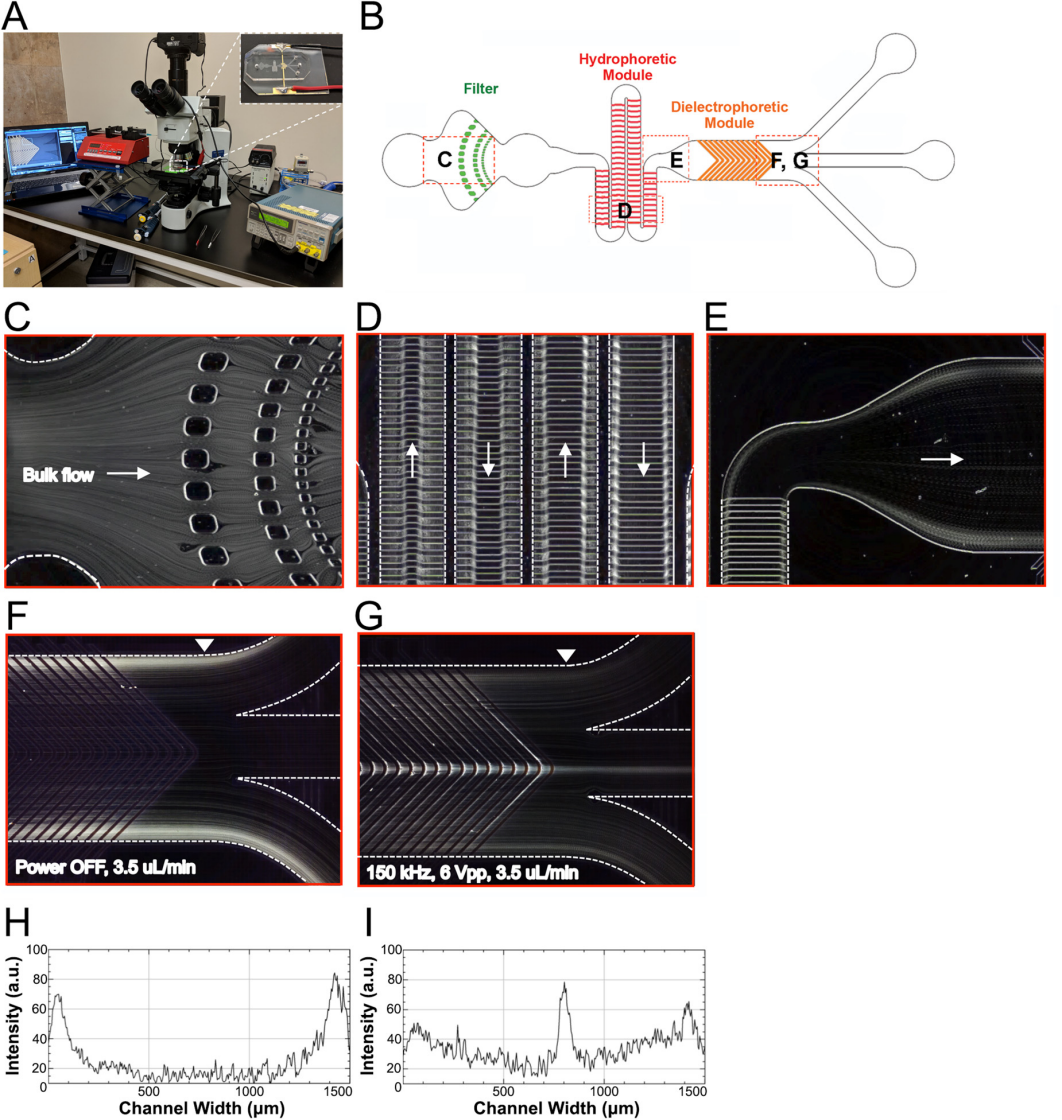
Functional analysis of the HOAPES device. (a) For sorting cells, the HOAPES device (inset) is mounted on the stage of an upright microscope to enable visualization of cells in the channel. The device is connected to a function generator for DEP and syringe pump for constant fluid flow. (b) Red dashed boxes on the HOAPES device schematic show the locations where images in (c)–(g) were taken. (c) The trajectories of mouse NSPCs in the HOAPES device were obtained from Z-projections (see the Materials and Methods section). Individual cells easily pass the PDMS pillars of the filter region designed to prevent cell clumps from entering the channel. (d) In the hydrophoretic module, cell trajectories show that cells are gradually directed into 2 streams along the channel edges. Arrows indicate the direction of fluid flow. (e) At the end of the hydrophoretic module, cells are in 2 streams along the channel edges as they enter the DEP module. Arrow indicates the direction of fluid flow. (f) When power to the DEP module is off, the cells remain in 2 streams along the channel edges and exit the two outer channels. (g) When power to the DEP module is on and electrodes are actuated at 150 kHz and 6 Vpp, a subset of cells are focused to the middle of the channel and exit via the inner channel. (h) Plot shows the signal intensity of cell trajectories across the channel width when power to the DEP module is off [image in (f)]. The arrowhead in (f) indicates the region of the channel across which the signal intensity was measured. Two peaks are evident along the channel edges. (i) Plot shows the signal intensity of cell trajectories across the channel width when power to the DEP module is on [(image in (g)]. The arrowhead in (g) indicates the region of the channel across which the signal intensity was measured. A peak is evident in the center of the channel, and the two peaks along the channel edges are lower than those in (h). Flow rate in (c)–(g) was 3.5 *μ*l/min.

We tested the functional operation of the HOAPES device by flowing through mouse NSPCs. The HOAPES device was mounted on a microscope stage to enable visualization of cell behavior during sorting [Fig. 3(a)]. Cells were loaded into the inlet port of the channel as described in the Materials and Methods section. The flow rate was first calibrated using a low cell concentration (1 × 10^6^cells/ml), an applied voltage of 6 Vpp (Simon et al., 2014; Lu et al., 2012), and a frequency at which more than 95% of the cells experienced pDEP and were focused to the inner channel (such as 1 MHz). Starting with a flow rate of 1 *μ*l/min, we gradually increased the flow rate and found that 3.5 *μ*l/min was the maximum rate that maintained more than 95% of the cells focused to the inner channel. We next optimized the cell concentration by switching to a lower frequency that could be used for sorting (such as 150 kHz) and gradually increasing the cell concentration starting at 1 × 10^6^cells/ml. We found that a cell concentration of 3 × 10^6^cells/ml could be effectively sorted while maintaining the purity (percentage of cells focused to the inner channel) obtained with lower cell concentrations. Based on these optimizations, we used a concentration of 3 × 10^6^ cells/ml and a flow rate of 3.5 *μ*l/min.

Cells entered the channel via bulk flow and encountered the filter region [Figs. 3(b) and 3(c)], which was designed to prevent the entry of large cell clumps that could clog the downstream channel. The filter effectively trapped large debris and cell aggregates but allowed fluid flow to continue through the channel so that sorting was not disrupted, increasing the utility of the sorter. Rarely, small cell clumps not trapped by the filter disrupted the function of the hydrophoretic module or the flow distribution at the channel outlets. The flow profile in the channel before and after the DEP module was closely monitored, and any runs with irregular flow due to these small cell clumps were discarded.

After passing the filter region, cells continued to spread across the channel width as they entered the hydrophoretic module. Cells in the hydrophoretic module were gradually directed to the outer edges of the channel through the action of PDMS microstructures on the channel ceiling that create hydrodynamic pressure gradients directing cell movement [Fig. 3(d) and Fig. S3 and Movie 2 in the supplementary material]. The location of the angles in the PDMS microstructures changes along the channel length, becoming successively further apart to direct cells toward channel edges [Figs. 1, 2, and 3(d) and Fig. S1 in the supplementary material]. At the end of the hydrophoretic module, the NSPCs were localized to two streams along the outer edges of the channel [Fig. 3(e)].

**FIG. 4.**
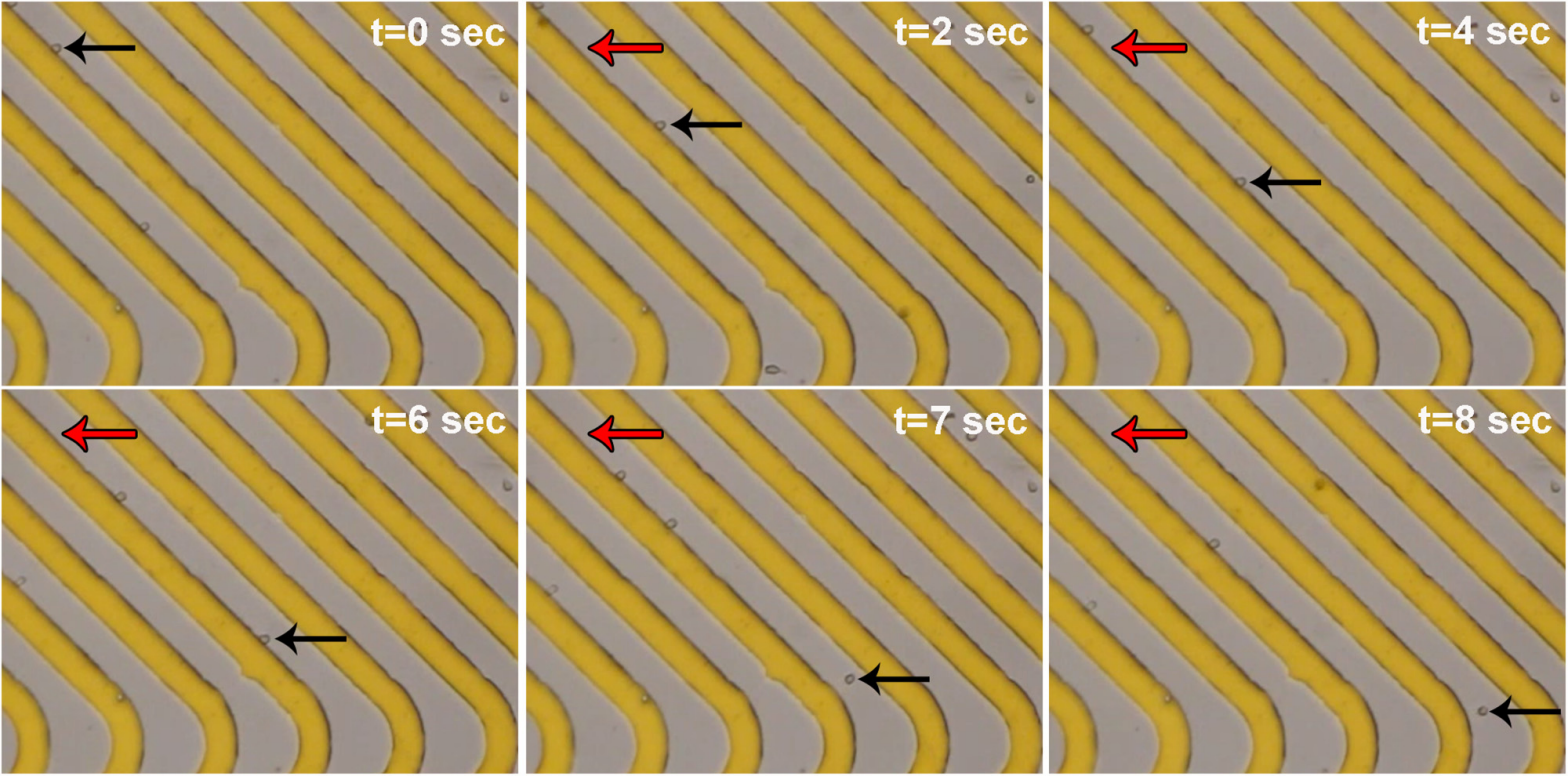
Movement of cells along electrodes in the DEP module. A time series of phase contrast images (10×) show cells moving along the slanted electrodes (gold) in the DEP module when electrodes were actuated at 150 kHz, 6 Vpp. One cell's position at each time point is shown by black arrows, while red arrows mark the starting position of that cell in the first panel. The last two panels (t = 7 s and t = 8 s) show the cell releasing from the electrode at the rounded tip and moving further down the channel.

Cells entered the DEP module in two streams along the channel edges. The electrodes in the DEP region are 35 *μ*m wide with 35 *μ*m gaps and are chevron-shaped, creating an angled electrode array pointing toward the center of the channel (Fig. 1 and Fig. S1 in the supplementary material). When the electrodes in the DEP module were not energized, the NSPCs remained along the channel edges and exited via the outer channels [Figs. 3(f) and 3(h)]. In contrast, when the electrodes were energized at an AC frequency at which a percentage of the cells experienced pDEP (150 kHz), the induced DEP force directed those cells along the electrodes, bringing them to the center of the channel [Figs. 3(g) and 3(i)]. Videos of NSPCs in the channel show their movement along the slanted electrodes (Fig. 4 and Fig. S3 and Movie 3 in the supplementary material). When cells reach the angled tips of the chevron electrode array, the reduction in induced DEP force allows them to be released from the electrode and travel down the channel (see the Device Design Principles section). The cells experiencing sufficient pDEP exit via the inner outlet. Varying the frequency of the applied electric field changes the percentage of cells in pDEP, enabling selection of subpopulations of cells.

### Experimental strategy for cell sorting in the HOAPES device

**FIG. 5.**
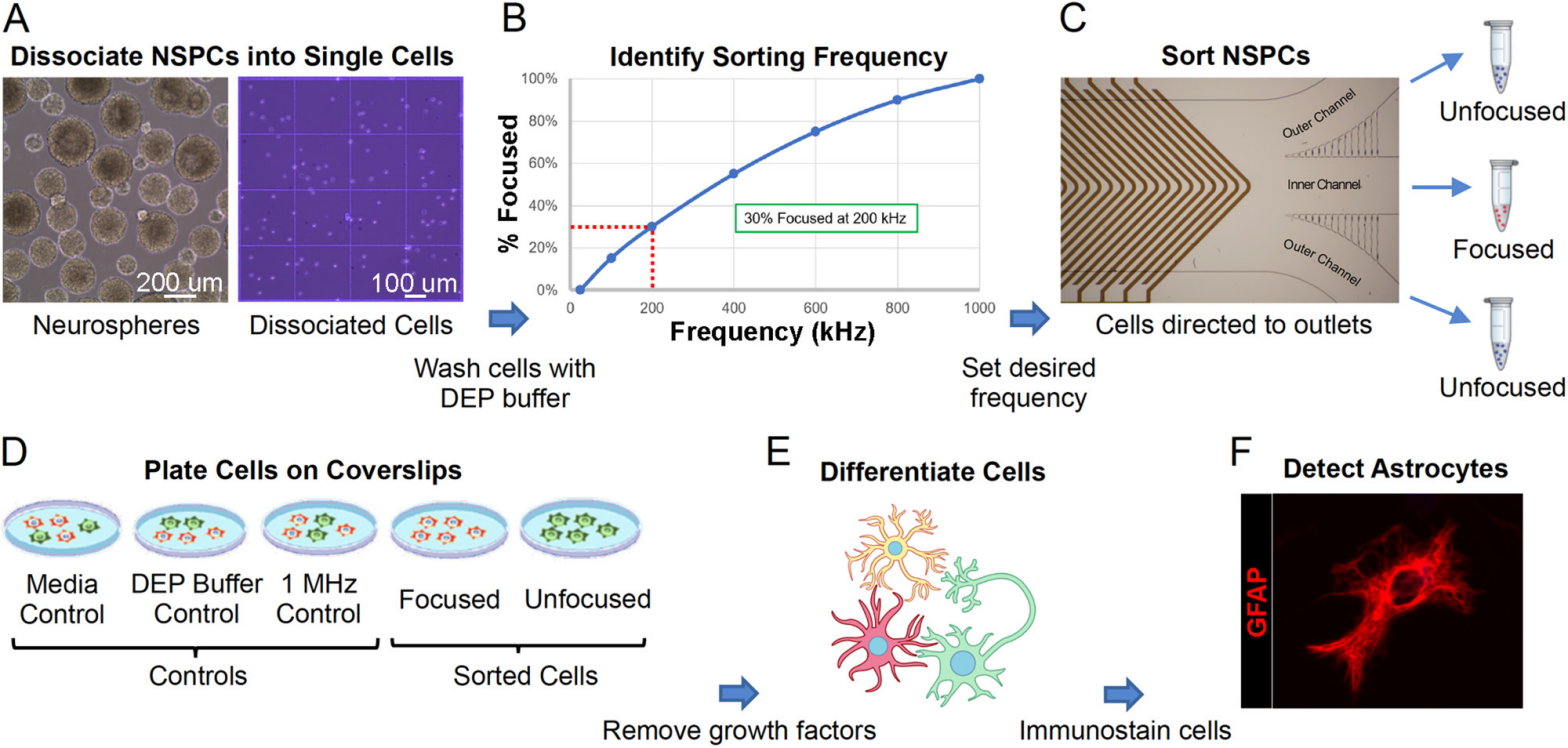
Experimental strategy for sorting mouse NSPCs with the HOAPES device. (a) Mouse NSPCs grow in suspension and form clusters of cells known as neurospheres (left). Neurospheres were dissociated to form single cells (right) and cells washed with the DEP buffer. (b) Cells in the DEP buffer were loaded into the HOAPES device, and the percentage of cells focused to the inner channel quantified across a range of frequencies. The resultant focusing curve (shown here as a schematic) was used to determine a sorting frequency targeting ∼30% of the cells. (c) Mouse NSPCs were sorted in the HOAPES device at the determined frequency and two populations of cells collected: those focused to the inner channel and those that remained unfocused and tracked to the outer channels. The unfocused cells from the two outer channels were pooled for further analysis. (d) Sorted cells (focused and unfocused) and controls (media, DEP buffer, and 1 MHz) were plated on glass coverslips for cellular differentiation and further analysis. (e) Cell differentiation was induced by the removal of growth factors from the culture media. Cells were differentiated for 3 days to allow formation of astrocytes. (f) Astrocytes in the differentiated cell samples were detected by immunostaining with antibodies that detect the astrocyte marker glial fibrillary acidic protein (GFAP, red). The image shows a differentiated astrocyte.

We utilized a standard workflow for each sorting experiment with the HOAPES device (Fig. 5). Mouse NSPCs are routinely grown in suspension and generate large clusters of cells termed neurospheres. Individual cells were dissociated from neurospheres for sorting in the HOAPES device [Fig. 5(a)]. Dissociated NSPCs were resuspended in a low conductivity DEP buffer solution for sorting, which we found previously does not harm NSPCs or alter their behavior (Flanagan et al., 2008; Lu et al., 2012). A focusing curve was generated for each batch of NSPCs to determine the appropriate sorting frequency since we find that the optimal frequency can vary slightly from batch to batch [Fig. 5(b)]. NSPCs were loaded into the HOAPES device and the desired frequency for sorting set such that approximately one third of the cells were focused to the inner outlet through pDEP (see the Determining Optimal Sorting Frequency section). The focused cells were collected from the inner outlet, and the unfocused cells along the channel edges were collected from the two outer channels and pooled [Fig. 5(c)]. Sorted cells were compared to several types of control cells: NSPCs grown in regular culture conditions (media control), NSPCs incubated in DEP buffer but not sorted (DEP buffer control), and NSPCs exposed to electric fields but not sorted (1 MHz control). The latter group was generated by setting a high frequency (1 MHz) at which all cells in the population experience pDEP and are focused to the inner channel. Since the goal of the sort was to enrich astrocyte-biased cells, we analyzed the fate of sorted cells and controls by differentiating the cells and quantifying astrocyte formation [Figs. 5(d)–5(f)].

### Determining optimal sorting frequency

**FIG. 6.**
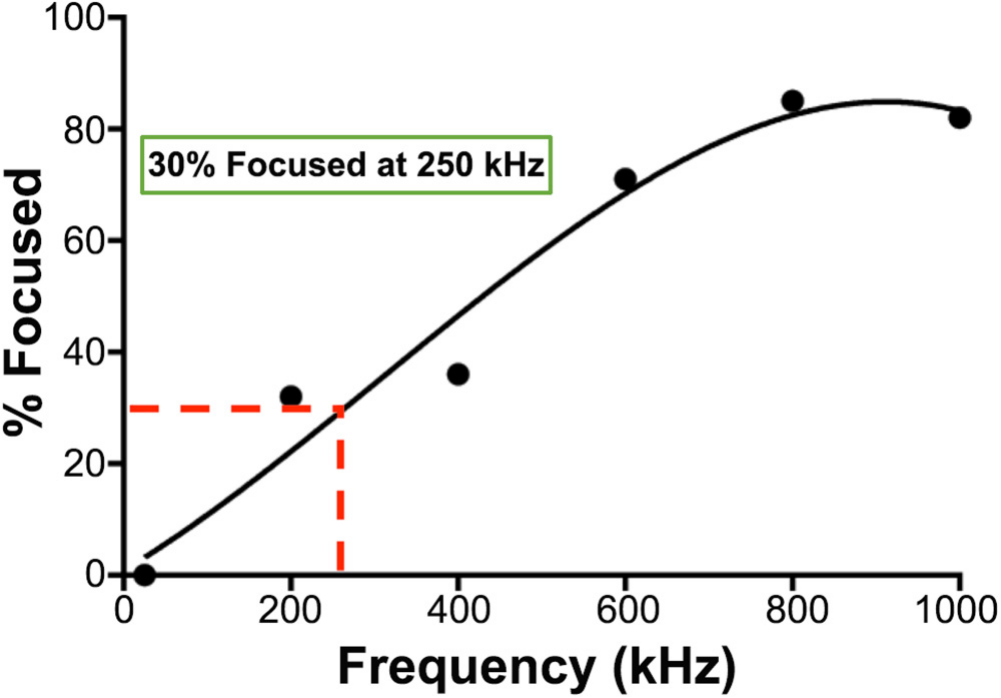
Representative focusing curve. A focusing curve from a mouse NSPC sorting experiment shows the gradual increase in the percentage of cells focused to the inner channel across increasing frequency. A best fit line was used to determine 250 kHz as the appropriate sorting frequency for this population.

We found previously that astrocyte-biased cells in NSPC populations experience pDEP at lower frequencies than do neuron-biased cells (Flanagan et al., 2008; Labeed et al., 2011; Nourse et al., 2014). Using a trap and release DEP-based sorting scheme, we determined that sorting cells at a frequency at which ∼30% of the cells experience pDEP led to the enrichment of astrocyte-biased cells (Simon et al., 2014). We tested a similar approach here, aiming to collect 30% of the cells experiencing pDEP at low frequencies. Since the HOAPES device incorporates fluid flow and continuous movement of cells along the electrode array, we experimentally determined the frequency needed to focus ∼30% of the cells to the inner channel outlet. We generated focusing curves for each batch of mouse NSPCs by measuring the percentage of cells focused to the inner channel outlet at a range of frequencies from 10 to 1000 kHz (example shown in Fig. 6). We found that the frequency needed to target 30% of the cells ranged from 136 to 250 kHz across 4 batches of mouse NSPCs, with an average of 184 kHz.

### Sorting NSPCs in HOAPES yielded both enriched and depleted cell populations

**FIG. 7.**
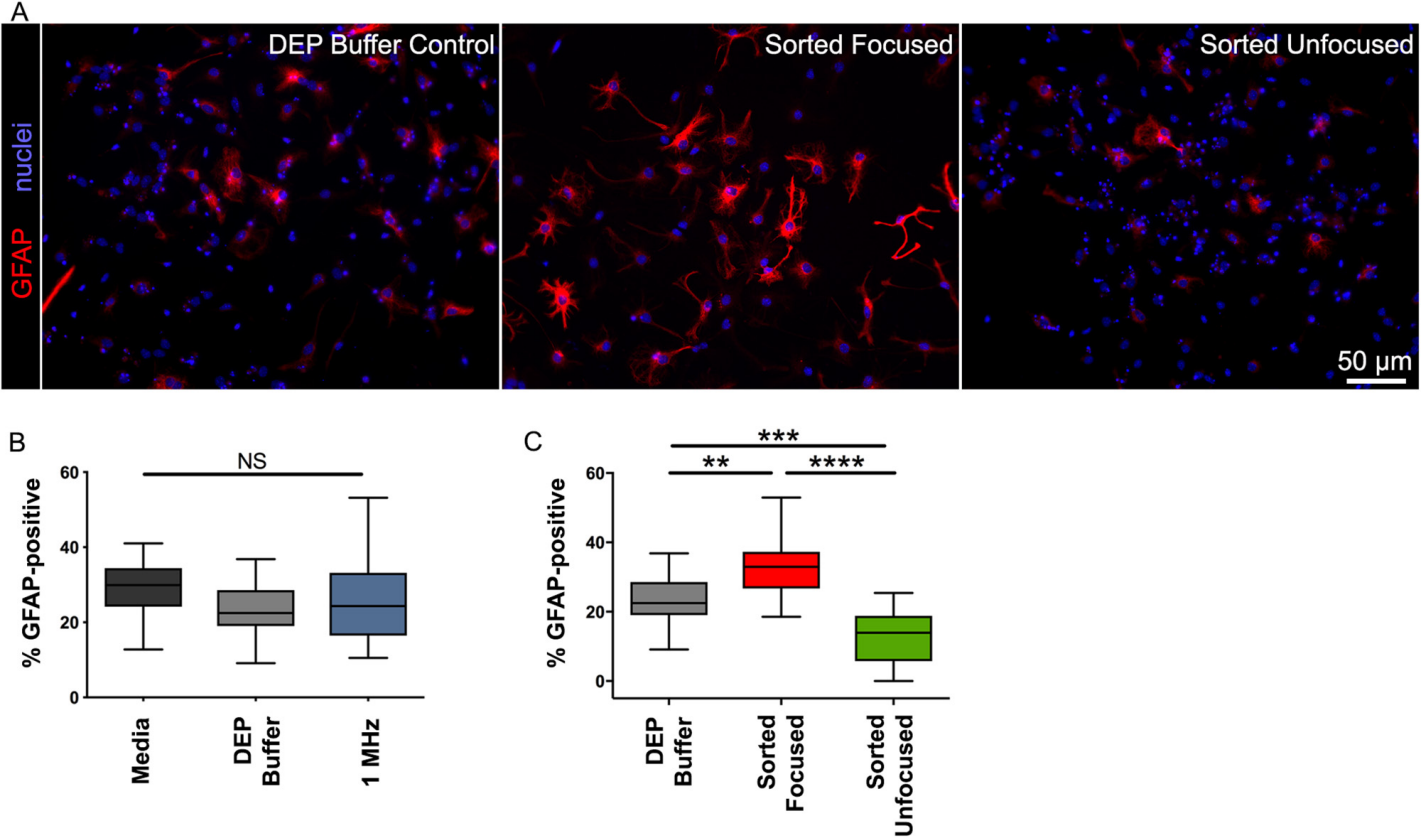
Astrocyte-biased cells are increased in focused and decreased in unfocused fractions. (a) Images of differentiated cells stained with antibody to GFAP (red) show that more cells in the focused population generated astrocytes. All cell nuclei were stained with Hoechst (blue). Scale bar 50 *μ*m. (b) Control cell populations did not differ in the percentage of cells that generated astrocytes. Controls included cells in regular culture media (Media), cells incubated in DEP buffer for the duration of the sorting experiment (DEP buffer), and cells sorted in HOAPES at a high frequency so that all cells are focused to the inner channel (1 MHz). (c) The percentage of cells forming astrocytes was significantly higher for focused cells and lower for unfocused cells. N = 4 independent biological repeats (one-way ANOVA with Tukey *post hoc* for multiple comparisons: **p < 0.01, ***p < 0.001, ****p < 0.0001, and NS, not significant).

We sorted mouse NSPCs in the HOAPES device at frequencies determined by focusing curves and differentiated the cells to assess astrocyte formation. Imaging of the differentiated cells showed robust astrocyte formation by cells focused to the inner channel [Fig. 7(a)]. We found no significant difference in the formation of astrocytes by the control cells (percentage GFAP-positive cells: 28.7 ± 1.6 Media, 23.9 ± 1.7 DEP Buffer, 26.1 ± 2.5 1 MHz, SEM; one-way ANOVA p = 0.25) [Fig. 7(b)]. Thus, cells incubated in the DEP buffer or exposed to 1 MHz electric fields did not differ from cells in the regular NSPC media in their ability to form astrocytes. These data corroborate our previous findings that DEP sorting of NSPCs does not change their fate potential (Lu et al., 2012).

We found significant differences in astrocyte formation by the sorted cells (percentage of GFAP-positive cells: 23.9 ± 1.7 DEP Buffer Control, 33.9 ± 2.2 Focused, 12.05 ± 1.7 unfocused, SEM; one-way ANOVA p < 0.0001) [Fig. 7(c)]. Astrocyte-biased NSPCs were significantly enriched in the focused sample, showing a 1.4-fold increase in cells forming astrocytes compared to control cells (p = 0.0015) [Fig. 7(c)]. Interestingly, astrocyte-biased cells were correspondingly depleted from unfocused samples collected from the outer exit channels [Fig. 7(c)]. Astrocyte-biased cells were reduced by half compared to controls (2-fold reduction) (p = 0.0001). The focused, enriched cells and unfocused, depleted cells differed from each other by 2.8-fold, providing two populations that differed more from each other than either population did from controls (p < 0.0001). The focused and unfocused cells give a higher degree of separation than that obtained with our previous sorting platforms. Thus, a significant advantage of the HOAPES device over other DEP sorting platforms is the generation of enriched and depleted cell populations in a single sort.

### Device reproducibility and throughput

**FIG. 8.**
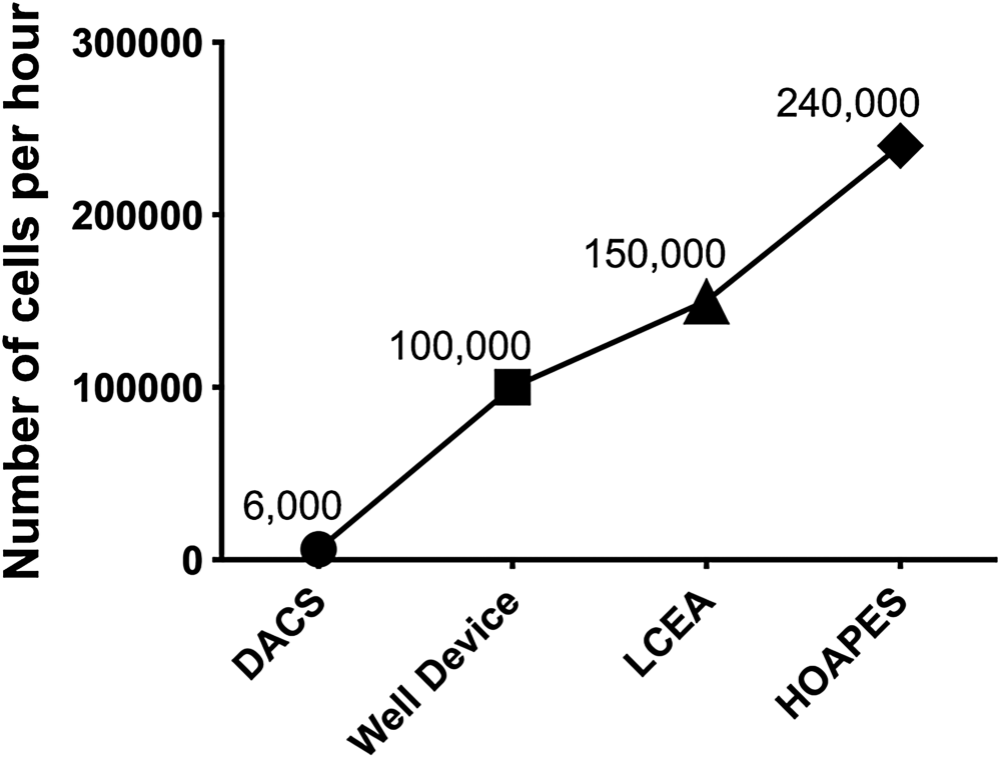
DEP-based sorting device throughput comparison. The actual throughput (No. of cells/h) is shown for several devices used to sort NSPCs.

The goal of the continuous sorting HOAPES device was to increase reproducibility and throughput of cell sorting. Sorting with the HOAPES device was more reproducible than sorting with our previous trap and release DEP-based sorters (Simon et al., 2014). This is likely due to the fact that the continuous sorter minimizes cell-cell interactions, eliminates residual flow, and reduces manual operation. The actual throughput of the HOAPES device is much higher than that of our previous DEP-based devices: the DACS device (Nourse et al., 2014), Well device (Simon et al., 2014), and LCEA device (Simon et al., 2014) (Fig. 8). Compared to our first generation trap and release DEP-based sorting device (DACS device) (Nourse et al. 2014), the HOAPES device delivers a 40-fold increase in the throughput of sorted cells.

## DISCUSSION

We created a microfluidic cell separation device incorporating hydrophoresis and DEP to continuously and rapidly sort cells. The hydrophoretic module efficiently directs cells to the edges of a microfluidic channel, lining up the cells for separation by the DEP module. The DEP module utilizes an array of slanted electrodes to target cells experiencing pDEP toward the channel center. Outlet channels separately collect cells focused by pDEP and those that remained unfocused. By providing two populations after sorting, focused and unfocused cells, the device generated enriched and depleted cell populations in a single sort. This was shown by sorting mouse NSPCs and determining that astrocyte-biased cells were increased in the focused fraction and decreased in the unfocused. The continuous sorting design greatly increased throughput over other DEP-based sorters. In sum, the new HOAPES device greatly improves label-free cell sorting by providing enriched and depleted cell samples in a single sort and increasing sorted cell throughput.

Professor Hsueh-Chia Chang has been a pioneer in the field of microfluidics and an inspiration to many who followed behind. Professor Chang's studies in the area of electrokinetics and microfluidic devices laid the groundwork for many of the design elements we incorporated into our device. In our previous DEP-based cell sorting devices, the cells enter the device as a disperse population spanning the channel width. However, in designing a continuous DEP-based sorter, we realized that directing cells to a particular starting point along the channel width would enable us to use the directed movement of cells along the channel width as a means of separation. Professor Chang's group used a similar strategy to separate microbial samples and red blood cells (Cheng et al., 2007; Cheng et al., 2009). Their devices used electrodes to induce nDEP to direct cells to the channel center. In our case, we hoped to direct cells to the channel outer edges and therefore employed a sheathless hydrophoretic cell aligner (Song and Choi, 2014; Song et al., 2015a; Lee et al., 2018b). We improved upon previous device designs by incorporating a gradual increase in the angle of the PDMS microstructures on the channel ceiling. This created a gradual movement of cells and, coupled with the length of the hydrophoretic module, resulted in seamless direction of cells to the outer edges of the channel (Figs. 1 and 3 and Movie 2 in the supplementary material). From there, the cells had a common starting point as they entered the DEP-based cell separation module of the device.

Electrodes for DEP-based cell sorting can be patterned in a wide array of geometries. When developing a DEP device for continuous cell sorting, our goal was to design electrodes to direct cell movement without trapping the cells in the channel. Toward a similar end, Professor Chang's group used a slanted electrode design to separate distinct cell populations in a continuous fashion. Cell movement was directed by induced nDEP forces, and the angle of the electrodes (and the frequency applied to the electrodes) determined whether each distinct cell type would track along the electrode or not (Kuczenski et al. 2011). The orientation of the electrodes in the *x*-*y* direction created “DEP gates” (electrode pairs at different orientations along the channel) that moved different cell types in different directions (Cheng et al., 2007; Kuczenski et al., 2011). This provided the basis of separation for distinct cell types. Negative DEP from slanted electrodes has also been combined with hydrophoresis to separate beads of different sizes or live and dead Chinese hamster ovary (CHO) cells (Yan et al., 2015). We sought to develop a continuous sorting system based on pDEP that could separate stem cells on the basis of fate potential (Flanagan et al., 2008; Labeed et al., 2011; Simon et al., 2014; Nourse et al., 2014).

We used a slanted electrode design for continuous sorting, but employed an array of parallel electrodes rather than a series of electrode pairs at different orientations along the channel. We created a chevron design of parallel electrodes since this design directs cells to the center of the channel (Hu et al., 2005). We found previously that pDEP forces effectively separate mammalian cells that are very similar to each other, particularly in terms of cell size and shape, so we designed our system around pDEP (Flanagan et al., 2008; Labeed et al., 2011; Simon et al., 2014; Nourse et al., 2014). Other designs have used slanted electrodes with pDEP to direct cell movement in a channel (Song et al., 2015b). However, in these devices, it was necessary to turn the electric field on and off in order for the cells to detach from electrode edges and proceed down the channel. We designed a chevron pattern with release points at the tips. This accomplished two goals—increasing the throughput by doubling the separation region and enabling cell release without the need to turn off the electric field. The observation of cells in the HOAPES device confirmed that induced pDEP forces were sufficient to direct cells along the slanted electrodes toward the channel center and that cells were released from electrode tips to continue moving down the channel (Figs. 3(g) and 4 and Movie 3 in the supplementary material). Our design facilitates continuous device operation, reduces possible error since the electric field need not be turned off at specific points in the separation, and increases throughput of DEP-based sorting.

The continuous cell sorting afforded by the HOAPES device overcomes several limitations of our earlier DEP-based sorting devices. The HOAPES device has only a single inlet, making fluid flow easier to control since it does not need to be balanced across multiple inlets. The filter in the HOAPES device prevents clogging by large cell clusters and debris, enabling continuous operation since experiments need not be stopped to remove channel blockages. Since cells are not trapped along electrodes, we avoid cell-cell interactions that might adversely impact the induced DEP forces acting on each cell. The lack of trapping also means that cells are exposed to electric fields for much shorter times (seconds rather than minutes), avoiding any potential toxicity in DEP electric fields (Lu et al., 2012). Additionally, DEP trap and release devices require turning the fluid pump on and off, which can result in residual fluid flow that can increase contamination with unsorted cells in the samples. In the HOAPES continuous flow design, residual flow is eliminated.

The HOAPES device increases throughput since the hydrodynamic forces acting on the cell aid separation by moving cells along the slanted electrodes. We optimized cell density and flow rate for mouse NSPCs in the device (3 × 10^6^ cells/ml and 3.5 *μ*l/min) and determined an actual throughput of 240 000 cells/h. This is a significant increase in throughput compared to our previous devices, which yielded a maximum actual throughput of 150 000 cells/h (Simon et al., 2014). This higher throughput, coupled with the fact that NSPCs can be expanded after sorting while maintaining enrichment, means that we can generate sufficient numbers of cells for most downstream applications, including stem cell transplantation (Simon et al., 2014). The theoretical maximum throughput of the HOAPES device operating at 3 × 10^6^cells/ml cell concentration, 3.5 *μ*l/min flow rate, and 6 Vpp with no cell losses or cell clumping and assuming a steady state separation would be 630 000 cells/h. Increasing the voltage during separation could potentially increase this throughput by allowing an increase in the flow rate or cell concentration, as long as the voltage increase does not harm the cells.

The HOAPES device utilizes AC DEP for cell separation, which provides flexibility for targeting different cell populations by varying the frequency of the applied electric field. We found previously that astrocyte-biased cells can be enriched from mouse NSPCs in low frequency DEP bands, while neuron-biased cells are in high frequency bands (Nourse et al., 2014). We set the sorting frequency for the experiments in the current study to target approximately one third of the cells in the population that experience pDEP at low frequencies. At those frequencies, we were able to generate cell populations enriched for and depleted of astrocyte-biased cells. The sorting frequency can be varied to target different cells in the population, resulting in changes to the percentage of cells directed to the inner channel outlet of the device. Thus, sorting can be performed at a wide range of frequencies to test for enrichment or depletion of cells of interest.

A significant improvement of the HOAPES device is the generation of enriched and depleted cell populations in a single sort. Many biological applications require the comparison of cells that may be quite similar to each other but differ in a key aspect. For stem cells, that key aspect can be fate, or what type of differentiated cell will be formed. Cells that are biased to form astrocytes are similar in size to other cell populations in NSPC cultures (Labeed et al., 2011; Nourse et al., 2014), but have very specific functions. Astrocyte-biased cells are of interest for transplantation in neurological diseases such as Amyotrophic Lateral Sclerosis (ALS), in which defective astrocyte function contributes to disease progression (Lepore et al., 2008; Yamanaka and Komine, 2018). The fact that the HOAPES device generates enriched and depleted cells enables experiments in which control, unsorted cells can be directly compared to populations making both more and fewer astrocytes. Comparison across a range of cells such as these, all derived from the same starting population of cells, reduces random sources of variation and allows analysis of cell properties directly related to the function of interest, in this case the ability to form astrocytes.

Cells in a heterogeneous population, such as stem cells, display a spectrum of inherent electrical properties. Our current device design generates two populations at the end of separation, but the distribution of electrical properties in stem cell populations suggests that separation into more bins, corresponding to tighter grouping of cells by electrical properties, could increase the purity of the separated cells. Future devices should take advantage of the full range of properties displayed by the cell population and collect more fractions to create finer separation.

DEP-based cell sorters are becoming increasingly relevant for a variety of cell biology and biomedical applications. We found previously that astrocyte- and neuron-biased cells do not differ significantly in cell size, but do differ in electrophysiological properties, namely, whole cell membrane capacitance (Labeed et al., 2011; Nourse et al., 2014). Thus, the HOAPES device is capable of separating cell types that are remarkably similar to each other, in this case subsets of NSPCs that are fated to different lineages and differ in membrane capacitance but not size. Recent studies demonstrate that the correlation of stem cell fate with cell electrophysiological properties and cell behavior in DEP is not limited to the neural stem cell lineage. Similar patterns hold true for embryonic/pluripotent stem cells, mesenchymal and adipose-derived stem cells, hematopoietic stem cells, and cancer stem cells (Lee et al., 2018a). Consequently, DEP-based sorting devices are relevant for isolation of cells destined to form particular types of differentiated cells from many starting populations of stem cells.

Sorting by DEP is applicable across a wide swath of biological and medical applications. DEP has sufficient resolution to distinguish similar populations of cells, including stimulated vs nonstimulated Jurkat cells (Pethig et al., 2002), subpopulations of human leukocytes (Yang et al., 1999; Vykoukal et al., 2009), red blood cells on the basis of ABO type (Srivastava et al., 2008), and breast cancer cells transfected with the neu oncogene (Cristofanilli et al., 2002). DEP analysis is capable of identifying apoptotic cells before traditional markers are expressed (Chin et al., 2006; Labeed et al., 2006; Mulhall et al. 2015), the efficacy of cancer treatments (Mahabadi et al., 2018), and the status of intracellular mitochondria (Rohani et al., 2017). The pathogenicity of microbes can be detected by DEP, enabling rapid detection of dangerous organisms (Fernandez et al., 2017). Further development of label-free DEP-based sorting devices will be critical for many areas of cell biology and medicine.

## SUPPLEMENTARY MATERIAL

See supplementary material for Fig. S1: HOAPES device dimensions, Fig. S2: Simulation of fluid flow at different heights in the channel, Movie 1: Video showing fluid simulation at various planes in the hydrophoretic module, Fig. S3: Cell movement through the hydrophoretic and DEP modules of the HOAPES device, Movie 2: Video showing movement of NSPCs through the hydrophoretic module, and Movie 3: Video showing movement of NSPCs through the DEP module.
